# Comparative effectiveness of interventions incorporating physical activity in individuals with comorbid low back pain and depressive symptoms: a systematic review with network meta-analysis

**DOI:** 10.1038/s41598-026-54397-0

**Published:** 2026-06-03

**Authors:** Nils Haller, Leonard Köbel, Larissa Schäffer, Jonathan Overath, Daniel Niederer

**Affiliations:** 1 Department of Sport Medicine, Rehabilitation and Disease Prevention, Johannes Gutenberg University Mainz, Mainz, Germany; 2 Department of Sport and Exercise Science, University of Salzburg, Salzburg, Austria; 3 Division of Exercise and Movement Science, Institute for Sport Science, University of Göttingen, Göttingen, Germany; 4 Department of Movement and Training Science, Faculty of Humanities and Social Sciences, Institute of Sport Science, University of Wuppertal, Wuppertal, Germany; 5 Institute for Occupational, Social and Environmental Medicine, Goethe University Frankfurt, Frankfurt am Main, Germany

**Keywords:** Exercise, Mental health, LBP, Chronic disease, Sports therapy, Rehabilitation, Patients, Diseases, Health care, Medical research, Psychology, Psychology

## Abstract

This systematic review with network meta-analysis aimed to identify the most effective intervention incorporating a substantial physical activity component in individuals with comorbid depressive symptoms and nonspecific chronic low back pain (LBP). A search of six databases (PubMed/Medline, PsycINFO, Web of Science Core Collection, EMBASE, CINAHL, CENTRAL) was conducted up to July 30, 2025. Randomised controlled trials on participants > 15 years with comorbid clinically relevant depressive symptoms (e.g., Beck Depression Inventory (BDI)-II > 13) and chronic (> 12 weeks) LBP were included. Interventions involved ≥ 33% active movement and lasted ≥ 2 weeks. Outcome-wise risk of bias (RoB) for the self-reported outcomes was assessed using the Cochrane RoB 2 tool. Primary outcomes were (i) changes in depressive symptoms (mandatory) and (ii) pain intensity, disability and quality of life, with each study required to include at least one of three additional outcomes. We calculated frequentist-based network meta-analysis. Of 2138 studies, five studies (*N* = 834, 50.1% female, mean age: 52.8 years) with eight treatments were included. Overall RoB ranged from low to some concerns. Yoga with education was most effective at mid-term follow-up (intervention duration closest to 12 weeks, k = 9), showing improvements in pain (SMD=−1.05 [95%CI=−1.38 to −0.72]) and clinically relevant improvements in depressive symptoms (SMD=−1.48 [95%CI=−1.81 to −1.14]), all vs. usual care. Antidepressant therapy with pain self-management (e.g., physical activity, relaxation, breathing) moderately affected depressive symptoms (SMD=−0.56 [95%CI=−0.60 to −0.53]) and pain (SMD=−0.53 [95%CI=−0.56 to −0.49]). At long-term follow-up, antidepressant therapy with pain self-management, and online- and mobile-based therapy showed superiority over usual care. The overall certainty of evidence across outcomes was low to very low. With very low certainty evidence, yoga combined with education yielded the most favourable effects across outcomes. However, these findings should be interpreted with caution and require adequately powered future trials. While other approaches showed small to moderate effects, their clinical relevance remains uncertain.

**PROSPERO 2024 ID**: CRD42024523604.

## Introduction

Major depression affects around 17% of the population over their lifetime^1^, while nonspecific low back pain affects over 80% of individuals at least once in their lives^2^. Consequently, both conditions rank among the most prevalent mental health^1^ or physical health^2^ issues, respectively. Individuals with low back pain or depression have a considerably higher risk of developing the other condition, when compared to those without either^3,4^. Chronic musculoskeletal pain is accompanied by comorbid depression in 30% to 50% of all cases, with both conditions negatively reinforcing each other^5^. Consequently, when both conditions occur as comorbidities, the combined treatment is of critical importance.

For chronic (> 12 weeks) low back pain (LBP), exercise therapy, when necessary combined with psychotherapeutic interventions such as cognitive behavioral therapy^6^, is recommended as first-line treatment^7–9^. Exercise interventions focusing on stabilization, such as motor control exercises, functional strengthening, Pilates, and sensorimotor training, have also shown favourable results in individuals with chronic LBP^10–12^.

For the treatment of major depression, exercise therapy is an effective adjunct to psychotherapy or pharmacological treatment^13,14^, receiving the highest recommendation level in national guidelines for unipolar depression as an adjunctive intervention^15^. Exercise is considered a low-risk intervention compared to conventional treatments, such as pharmacotherapy, and does not require the waiting periods often associated with psychotherapeutic interventions^16^. Endurance training is particularly effective for the treatment of depression, while a combination of endurance and strength training, as well as mind-body approaches, have also been shown to reduce depressive symptoms^13,17,18^. Thus, exercise modalities recommended for depression are not necessarily identical to those commonly recommended for LBP.

Given that many individuals experience LBP and depression not in isolation but as comorbidities^19,20^, it remains unclear which exercise-based strategies are most appropriate when both conditions coexist. An intervention effective for LBP alone may not sufficiently address depressive symptoms, and vice versa. A network meta-analysis allows simultaneous comparison and ranking of multiple interventions, even when direct comparisons are limited. Thus, we have conducted a systematic review with network meta-analysis to assess the comparative effectiveness of interventions that include a substantial physical activity component in individuals with comorbid depression and LBP. The objective was to identify therapeutic approaches that address both conditions simultaneously and to evaluate their effectiveness in terms of improving pain, disability, depressive symptoms, and quality of life in this population.

## Methods

### Protocol and registration

This systematic review with network meta-analysis was performed with an a priori defined protocol which is published in the PROSPERO database (ID: CRD42024523604). A deviation from the protocol occurred during review processing: To ensure comparability between study arms and interventions, an assessment and rating of transitivity was made a posteriori.

The study was conducted in accordance with the PRISMA extension statement incorporating network meta-analyses as well as the recommendations of the Preferred Reporting Items for Systematic Reviews and Meta-Analyses and the PERSiST guidance (implementing PRISMA in Exercise, Rehabilitation, Sport medicine and Sports science^21^). The completed PRISMA NMA checklist can be found in the Appendix 1.

### Eligibility criteria

We included randomised controlled trials (RCTs), published in English or German. The inclusion and exclusion criteria followed the participants – interventions – comparator – outcomes (PICO) scheme^22^.

#### Population

Persons (> 15 years of age) with chronic (> 12 weeks duration) nonspecific LBP (i.e., “back pain with no known underlying pathology”^23^) and meeting clinical cut-offs for at least mild depression (e.g. >13 on the Beck depression inventory-II^24^) were included. Studies assessing only one of these conditions, i.e., either individuals with LBP or such with depressive symptoms, were excluded. Trials in which only one condition (either LBP or depressive symptoms) was required for inclusion were excluded. Furthermore, if depressive symptoms or LBP was only assessed as a secondary outcome, studies were only eligible if all participants in the study fulfilled both criteria.

#### Intervention

Physical activity interventions for depressive symptoms and LBP as monotherapy or in combination with other treatments were included. To ensure a meaningful physical activity dose in multi-component programs typical for this population, we required that ≥ 33% of the intervention time comprised active movement (as physical activity is often embedded within broader behavioural or multidisciplinary programs^15,25,26^) with a duration of at least 2 weeks (to avoid excluding potentially relevant trials). There were no restrictions on any method of implementation. Additional therapies (e.g., behavioural therapy, self-system therapy, education) were permitted. These interventions were classified as comparators unless they were combined with exercise/physical activity for depressive symptoms and LBP.

#### Comparators

All controls (active and passive) such as non-exercise interventions, real/true control, usual care, other exercise interventions, psychological or cognitive interventions, placebo, education, or medication were included. Pre- and post-measures were required for both diagnoses, i.e., depressive symptoms and LBP.

#### Outcomes

Primary outcomes were (i) changes in depressive symptoms (mandatory) and (ii) pain intensity, disability and quality of life, with each study required to include at least one of these three additional outcomes. Depressive symptoms had to be measured with any clinically validated tool. Pain intensity, disability and quality of life must be measured using standardized scales in each respective domain. Studies that assessed only one of the required outcomes at baseline were included only in the analysis of the other eligible outcomes, provided they met all other inclusion criteria.

### Data sources and electronic searches

Our search strategy was conducted in accordance with the PRESS guideline statement of electronic search strategies^27^. Six scientific online databases (Pubmed/Medline, PsycINFO, Web of Science Core Collection, Excerpta Medica Database, Cumulative Index to Nursing and Allied Health Literature, Cochrane Central Register of Controlled Trials) were searched from their inception to July 30, 2025. To identify potentially unpublished studies (e.g., preprints or early online versions) that might not yet be indexed in traditional databases we searched Google Scholar considering the first 300 hits. We also performed a forward citation tracking; the reference lists of the included studies were manually searched. We only included randomised controlled trials (RCTs) published in German or English language. The full search strategy can be found in Appendix 2.

### Study selection

Three reviewers (LS, JO, LK) independently screened all titles and abstracts of the search results to identify relevant articles. Subsequently, full texts were evaluated with respect to the inclusion and exclusion criteria. Disagreements were resolved through discussion, involving the entire review team when necessary.

### Data extraction

Data extraction was performed independently by three reviewers. Missing data were requested via email from the corresponding authors with two reminders, if needed. General information about the study such as authors, year, and country of publication were extracted first. Additional information regarding potential effect modifiers were collected.

#### Participants

We extracted key study characteristics, including participant numbers, average age, gender distribution, and pain duration.

#### Interventions

All required information about the intervention was extracted: detailed type(s) of physical activity/exercise(s), short description of the intervention, intervention mode, delivery format (supervised, group), dose (session length, sessions per day, frequency, program duration).

#### Outcomes

We extracted the prioritized outcome of each outcome dimension: depressive symptoms as well as pain intensity, disability, and quality of life, if available. Depressive symptoms and at least one of the three latter outcomes of interest had to be reported for inclusion into the network meta-analysis.

Our primary endpoint was the measurement closest to 12 weeks after randomization (usually assessed immediately after the intervention) referred to as “mid-term”. Only measurements assessed between 8 and 26 weeks (i.e., 6 months) after randomization were included in the mid-term analyses. In addition, and if available, data assessed at a “short” (2 to 6 weeks, “short-term”) and a “long” follow-up time point (> 26 weeks, “long-term”) were considered eligible.

#### Study risk of bias assessment

The Cochrane Risk of Bias tool 2 (RoB 2)^28^ was used by three independent reviewers for assessing the risk of bias of the individual studies’ outcomes of interest. All self-reported outcomes (i.e., pain intensity, disability, and quality of life) were rated as having the same risk to be influenced by any domain of bias. The following RoB 2 domains were assessed for depressive symptoms, pain intensity, and disability: risk of bias arising from the randomisation process or from deviations from the intended intervention, missing outcome data, measurement of the outcome and selection of the reported results. Biases were rated as high, low, and unknown risk/some concerns, from which an overall risk of bias rating was built.

### Data analysis

The primary analyses consisted of descriptive statistics and network meta-analyses for the four outcome parameters at post-intervention and follow-up. Data preparation and analyses were performed via Microsoft Excel 2016 for Windows (Microsoft Corporation, Redmond) and R Studio (Version 4.4.1, the R Foundation for Statistical Computing, Boston, MA) using the R packages *netmeta*,* pcnetmeta*,* metafor* and *dmetar*. Alpha level of significance was set, a priori, to *p* < 0.05. The full analysis code is displayed in Appendix 3.

#### Descriptive analyses and review of network geometry

Descriptive analyses reported the total number of participants, the number of treatment classes, and the pairwise comparisons (both design-specific and overall comparisons). Additionally, the network geometry was visualized using network plots. In this representation, each intervention class is depicted as a node, with the size of the node corresponding to the number of patients included in that intervention class. Direct within-study comparisons are illustrated by lines connecting the nodes. The thickness of these lines reflects the number of studies contributing to the comparison, with thicker lines indicating a higher number of included studies.

#### Effect measure

To compare the different treatments, measures of central tendency (mean or median) and standard deviation (SD) were utilized. When SD was not provided in the article, but other measures of dispersion were available, it was calculated or estimated based on Cochrane Collaboration guidelines^29^. Individual effect sizes and their variance were determined using between treatment pre- to post-differences for each comparison, expressed as standardized mean differences (SMD; Hedges’ g). If studies reported multiple outcome domains and measurement points, effect sizes were calculated for each domain and time point. Clinical relevance was assumed when the confidence interval of the effect estimate exceeded the predefined threshold for clinical relevance. To facilitate interpretation, the mean difference was recalculated by multiplying the SMD by the pooled standard deviation of outcomes across trials^30^. Clinical relevance was evaluated using thresholds: SMD of 0.5 for depressive symptoms (used as a distribution-based proxy for minimal important change (≈ 0.5 standard deviations^30,31^;) and mean differences of 1.5 cm in pain intensity and 1.0 cm in disability on the recalculated 10 cm visual analogue scale^32^.

#### Transitivity assumptions

To assess the comparability between treatment arms, numerous transitivity assumption assessments were undertaken. First, joint randomisability^33^ was assured using participant and intervention inclusion criteria leading to a homogeneous sample in terms of the indication for the interventions. Second, the following potential effect modifiers were defined and assessed between trials: baseline depressive symptoms and baseline pain intensity. The distribution and values of these potential effect modifiers were subsequently compared visually for pairwise treatment comparisons using box plots^34^.

#### Network meta-analysis and treatment rankings

A random-effects model was used to pool standardized mean differences (SMDs, Hedges’ g) with corresponding 95% confidence intervals (CI) as deviations from mean SMDs. The network meta-analysis followed a frequentist approach, treating each specific intervention type and comparator as individual nodes while accounting for multi-arm studies. All treatments were evaluated against the predefined reference “care as usual”, which often serves as the conventional therapy in RCTs. The P-score was applied to rank interventions, summarizing their effectiveness on a scale from 0 to 1^35^. Higher P-scores indicated a higher likelihood of being the most effective treatment. However, interventions with high rankings but no significant results were excluded from the final interpretation of optimal treatment options. Contrast-based forest plots were generated, presenting SMDs and 95% CIs, with treatments ordered in descending P-scores to facilitate comparison.

#### Assessment of inconsistency

We assessed global heterogeneity and inconsistency in our network model using the tau² and I² statistics. To evaluate significant within-design heterogeneity and between-design inconsistency, we employed Q statistics. Furthermore, we examined local consistency within the network through the net-splitting method, which separates network estimates into components derived from direct and indirect evidence^36^.

#### Publication bias

Publication bias was tested via comparison-adjusted Egger’s regression test for the screening of comparison-adjusted Funnel plot asymmetry^37^. Analyses were carried out for each outcome at mid-term, but only if > 10 direct treatment comparisons existed. Treatment order was fixed as: multimodal active treatments > unimodal active treatments > passive treatments > controls.

#### Sensitivity and subgroup analyses

We considered the severity of symptoms as the most important effect modifier. In the sensitivity analyses, only studies with participants rated as having at least moderate depressive symptoms (e.g., BDI-II> 19^38^), and clinically relevant pain intensities (> 3 on a 0–10 visual analogue scale)^39^ were included.

#### Certainty of the evidence

The GRADE (Grading of Recommendations, Assessment, Development, and Evaluation) framework, specifically updated for network meta-analyses^40^, was utilized to evaluate the certainty of evidence. This assessment accounted for study design, risk of bias, imprecision, inconsistency, indirectness, and publication bias.

## Results

### Study search, selection, and inclusion

From 2138 identified studies, we included 5 RCTs^41–45^ in the review. The included trials contained 834 individuals in total. All included RCTs provided sufficient data for the network meta-analysis. The detailed study search and selection process can be found in Appendix 4, and detailed reasons for excluding a trial in the review in Appendix 5. Descriptions of the treatments and the number of study arms with each treatment class can be found in Table 1. The mean intervention duration was 19 weeks.

**Table 1:** Treatment categories characteristics.

Treatment category	Function in the NMA	Description	No of nodes available for NMA
Antidepressant therapy and pain self- management^41^	Intervention	Combined antidepressant therapy and pain self-management program: the intervention was conducted in three phases. The first phase was an antidepressant optimization for 12 weeks, during which medications were adjusted to ensure effective treatment of depression. In the second phase, which was a 12-week pain self-management program, various physical activities were used in the sessions (e.g. stretching, strengthening and walking) alongside goal setting and behavioural changes. In the final phase, a six-month continuation period, participants were encouraged to maintain behavioral changes including the previously established physical activity behaviors^46^.	1
Online- and mobile- based therapy (Cognitive behavioral therapy^44^)	Intervention	“BackCare-D” is a cognitive behavioural therapy-based intervention combined with a physical activity online intervention, focusing on psychoeducation, physical activity, behavior activation, pain coping, and communication, which has been used in several studies. It involves addressing activity-related barriers and setting graded, everyday activity goals, individual physical exercises, using a personalized dosage over a period of up to 52 weeks. The intervention consisted of six core sessions, of which at least two targeted physical activity (e.g., development and implementation of an activity plan and addressing the relationship between physical activity and back pain), resulting in ≥ 33% physical activity component. In addition, three optional modules addressed sleep, partnership and sexuality, and return-to-work^47,48^. Participants had access to continuous online support throughout the intervention. Participants were advised to complete at least one session per week, which took an average of 54 min.	1
Yoga with education^45^	Intervention	Yoga combined with education program (8 weeks, two sessions per week) included education on spine biomechanics, breathing exercises, static and dynamic postures, Yoga Nidra, and Vipassana meditation. Sessions focused on relaxation, body awareness, and mindfulness, guided by an experienced yoga teacher. Of the 75 min per session, 60 min consisted of active yoga practice.	1
Education^42,45^	Comparator	LBP education sessions utilized a structured presentation and discussion format to educate participants on chronic LBP, covering its nature, diagnosis, treatment, and prevention techniques, and were delivered by experienced nurses or provided as a pamphlet.	2
Behavioral activation therapy^43^	Intervention	Behavioral activation therapy for depression (BATD) over 8 weeks aims to increase physical activity, improve sleep and reduce stress by engaging in positive, value-based activities. BATD focuses on daily monitoring of activities, physical exercises, planning valued behaviours, and social support to improve mood, thoughts, and life quality. Physical activity represented one of the three main components of the intervention and was addressed both during the weekly 90-minute sessions and through self-guided activities between sessions. Based on the available intervention description, physical activity constituted a substantial component of the programme and met our predefined ≥33% criterion.	1
Acceptance and commitment therapy^43^	Comparator	Acceptance and commitment therapy (ACT) (8 weeks with one session per week for one and a half hour) promotes acceptance of unwanted experiences while encouraging value-based, goal-directed actions. It aims to enhance psychological flexibility. ACT targets: openness, awareness, and active engagement.	1
Self system therapy^42^	Comparator	Self-system therapy (SST) is a structured, short-term therapy for depression, focusing on self-regulation and promotion-focused goals. It involves 12 weekly 90-minute sessions using behavioural activation, goal setting, and pain management techniques.	1
Care as usual^41–44^	Comparator	In care as usual, patients either continued their existing treatment or were placed on a waiting list.	4

NMA, network meta-analysis.

### Risk of bias at the outcome level

**Fig. 1:**
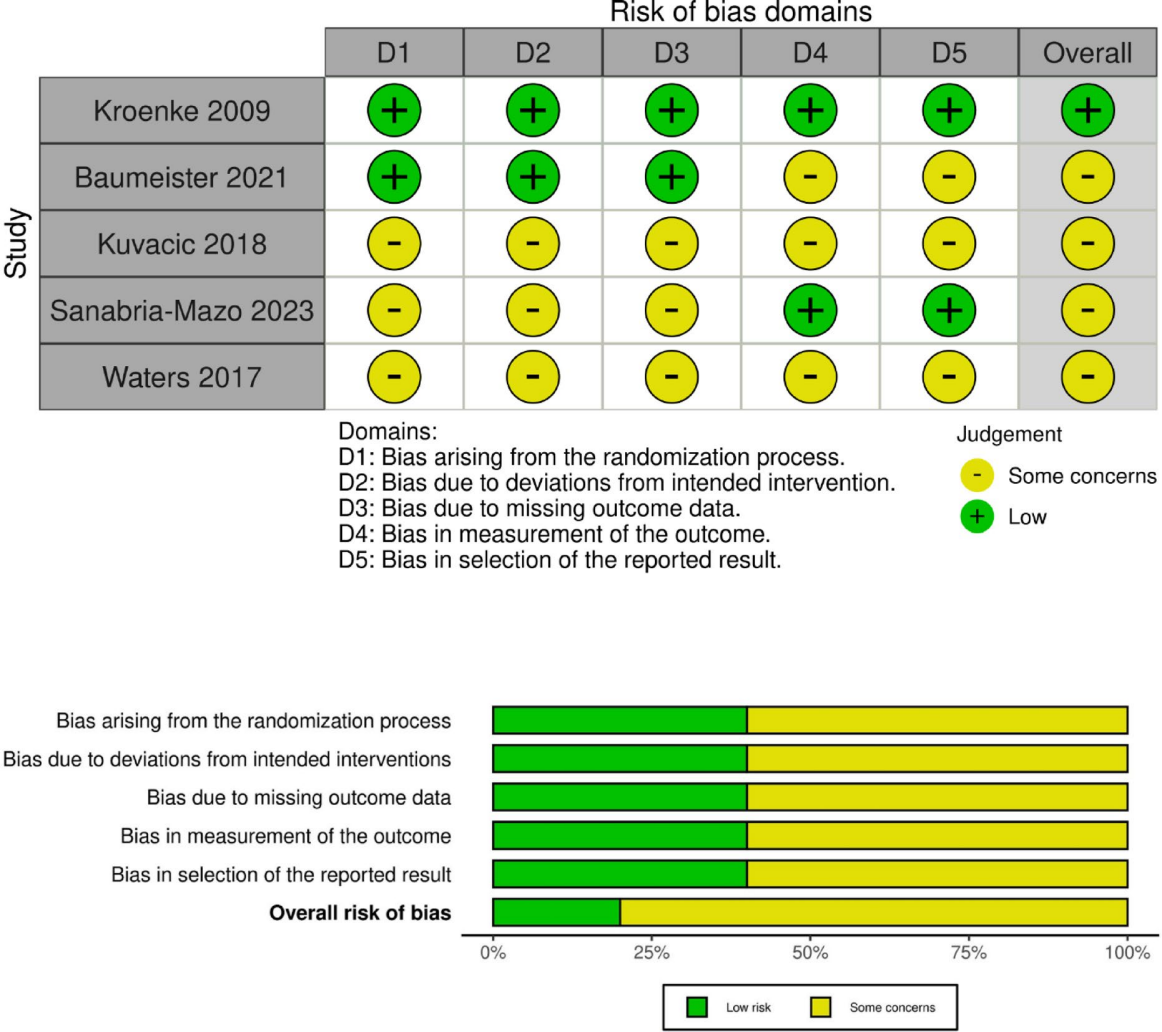
Risk of bias ratings. Upper part: Traffic light plot to display each domain’s risk of bias assessment for the outcomes of the included trials. Bottom part: summary plot to display the risk of bias distribution of the risk of bias domains across the included trials.

Out of the five included studies, only one had a low overall risk of bias in assessing the outcomes, while some concerns were found in the remaining four studies. The distribution of ratings across individual domains and overall bias, along with detailed risk of bias assessments for each study, are presented in Fig. 1.

### Characteristics of the included trials and treatments

Study characteristics are presented in Appendix 6. Among the 834 included patients (mean age: 52.8 years, SD: 10.5) in the network meta-analysis, 50.1% of the participants were female. Overall, 18% of participants had mild depressive symptoms, 54% had moderate depressive symptoms, and 28% had moderate-to-severe depressive symptoms. All participants suffered from chronic LBP with a mean duration of LBP of 10.7 (SD: 8.3) years. Of the eight treatment classes included in the network meta-analysis, four were specific active exercise or physical activity interventions. Two treatment classes were active comparators (self-system therapy, acceptance and commitment therapy), another two were considered passive controls (treatment as usual, education). The duration of the intervention ranged from eight^43,45^ to 52 weeks^41^, with session frequency ranging from 0.5 to two sessions per week. Overall, the reporting of detailed information about the characteristics of the interventions was inconsistent. An overview of all intervention classes with definitions and their assignments to either exercise or comparator is provided in Table 1.

### Network meta-analyses

#### Main outcomes: mid-term effects

Due to a lack of data based on the study designs in the included trials, no short-term analyses were conducted. In the main mid-term analysis, five trials^41–45^ were included for pain intensity, five for depressive symptoms^41–45^, and three for disability^42,44,45^. No trial was excluded due to network connectivity. A total of nine pairwise comparisons were performed for pain intensity and depressive symptoms, and five comparisons for disability.

**Fig. 2:**
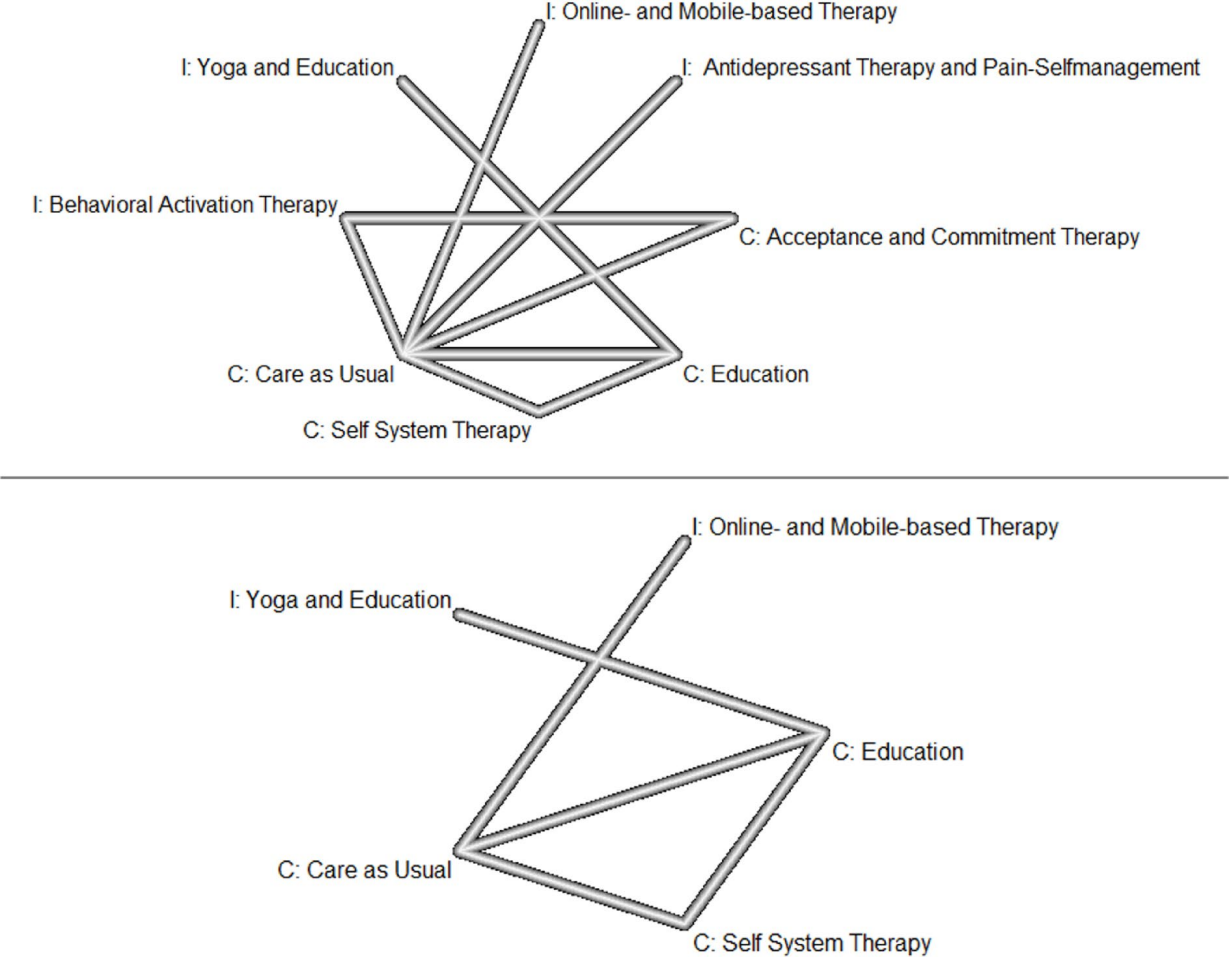
Network plot for pain intensity and depressive symptoms (upper part), and disability (bottom part). All interventions, comparators, and controls are displayed. The thickness of the lines indicates the number of the direct comparisons. I, interventions, C, controls/comparators.

Treatment-adjusted Egger’s test for funnel plot asymmetry was not calculated due to the low number of comparisons. For disability at mid-term, the number of studies was too low to assess publication bias. All networks were poorly connected (pain intensity (9 of 28 possible direct comparisons, 32%), depressive symptoms (9 of 28 possible direct comparisons, 32%), disability (5 of 10 possible direct comparisons, 50%)). Accordingly, a major proportion of the comparisons was based on indirect evidence. The corresponding network plots are depicted in Fig. 2.

Heterogeneity (within designs) and inconsistency (between designs) could not be calculated due to the small sample size. All evidence was either derived from direct or from indirect evidence. Thus, no indirect vs. direct evidence comparisons were made.

**Fig. 3:**
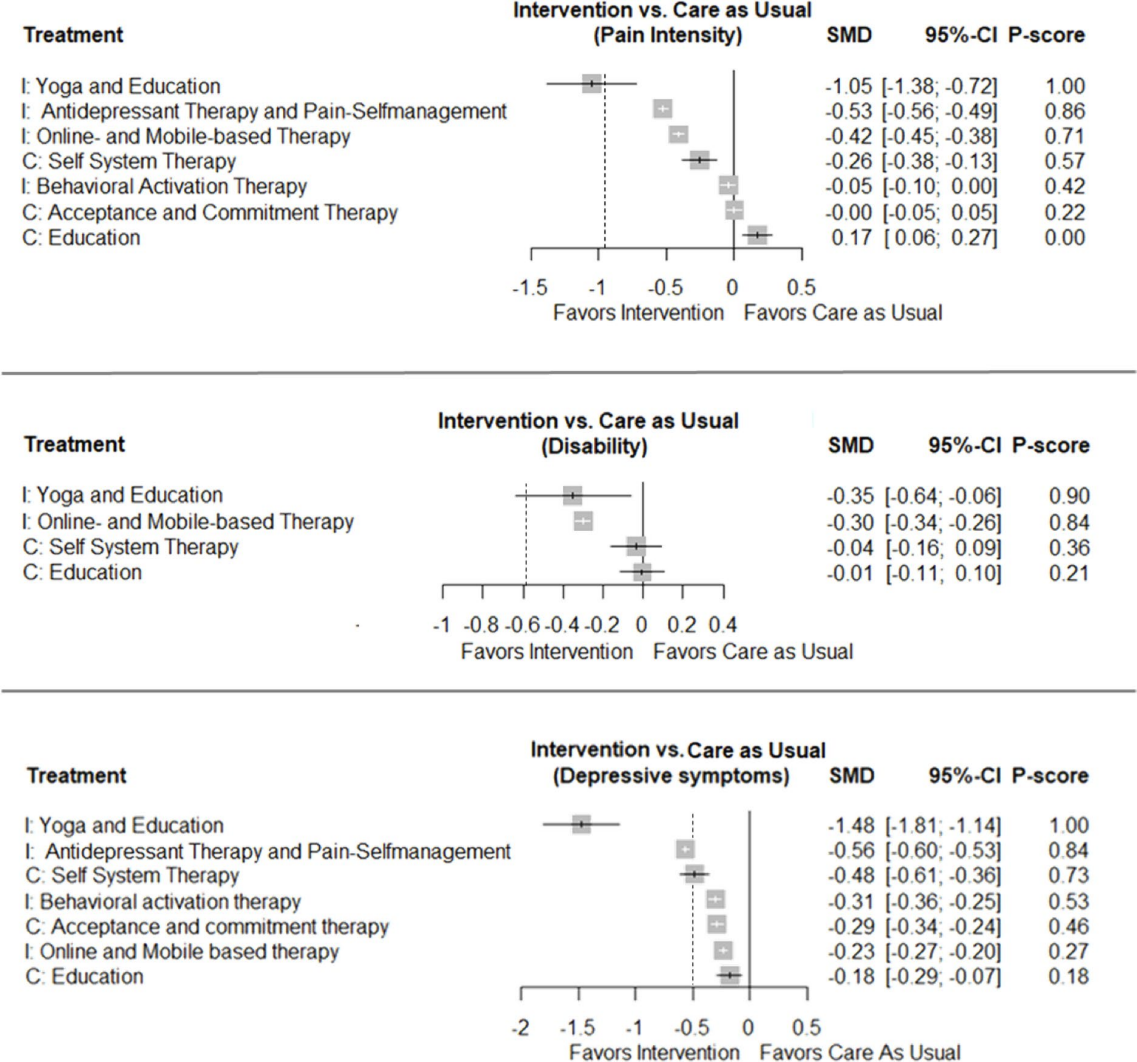
Forest plot for pain intensity (upper part), disability (mid part), depressive symptoms (bottom part), at mid-term. Each intervention, control, and comparator are compared to usual care. The vertical dotted lines indicate the clinical relevance thresholds. I, interventions, C, controls/comparators.

Figure 3 displays the network comparisons of all interventions, comparators, and the usual care groups. With very low certainty evidence, yoga with education was the most effective treatment and superior to usual care for pain intensity (SMD: -1.05, 95% CI: -1.38 to -0.72), depressive symptoms (SMD: -1.48, 95% CI: -1.81 to -1.14), and disability (SMD: -0.35, 95% CI: -0.64 to -0.06) reduction. The effect of yoga with education on depressive symptoms reached the threshold for clinical relevance. For pain intensity, the clinically not relevant superiority of antidepressant therapy and pain self-management (SMD: -0.53, 95% CI: -0.56 to -0.49) and of online- and mobile- based therapy (SMD: -0.42, 95% CI: -0.45 to -0.38) over usual care was further given with low certainty evidence. Low certainty evidence also supports the clinically not relevant superiority of all investigated interventions over usual care in terms of depressive symptom reduction. Beyond yoga with education (SMD: -0.35, 95% CI: -0.64 to -0.06), only online- and mobile-based therapy (SMD: -0.3, 95% CI: -0.34 to -0.26) was superior in reducing disability when compared to usual care (with low certainty evidence and clinically not relevant). The SMDs of the pairwise comparisons (for both direct and indirect evidence) can be found in Appendix 7. The detailed certainty of the evidence ratings including reasons for downgrading can be found in Appendix 13.

As all effects are estimated by direct or indirect effect estimation (no mixed evidence), no net splitting comparisons were performed. The direct evidence proportions are displayed in Appendix 8. Overall, the path length was *n* = 3 for 4 comparisons (pain intensity and depressive symptoms), and for 1 comparison (disability).

#### Secondary outcomes: effects at long-term follow-up

In the analysis for pain intensity at long-term, four trials were included^41–44^. For depressive symptoms, we could include four trials and for disability, three trials were included. For quality of life, two trials were eligible. No trial had to be excluded due to network connectivity. Heterogeneity (within designs) and inconsistency (between designs) could not be calculated due to the small sample size. Treatment-adjusted Egger’s test for funnel plot asymmetry was not calculated due to the low number of comparisons.

All evidence was either derived from direct or from indirect evidence. Thus, no indirect vs. direct evidence comparisons were made. Eight (pain intensity), eight (depressive symptoms), five (disability), and three (quality of life) pairwise comparisons were conducted. The corresponding network plots are depicted in Appendix 9. Appendix 10 displays comparisons of all interventions and the usual care groups at long-term. With low certainty evidence, antidepressant therapy and pain self-management (all outcomes), online- and mobile-based therapy (all outcomes), self-system therapy (pain and depressive symptoms), education (depressive symptoms) as well as acceptance and commitment therapy (pain and depressive symptoms) were superior to usual care. The detailed certainty of the evidence ratings including reasons for downgrading can be found in Appendix 13.

#### Transitivity and sensitivity analyses

Transitivity could not be statistically assessed due to the fact that each direct treatment comparison occurred only once. Descriptively, transitivity may not be given in baseline pain intensity and depressive symptoms. Thus, we performed a sensitivity analysis excluding studies with low baseline values in the effect modifiers pain intensity and depressive symptoms. In the resulting sensitivity for pain intensity and for depressive symptoms, we could include three trials. No trial had to be excluded due to network connectivity. Heterogeneity (within designs) and inconsistency (between designs) could not be calculated due to the small sample size. Treatment-adjusted Egger’s test for funnel plot asymmetry was not calculated due to the low number of comparisons. All evidence was either derived from direct or from indirect evidence. Thus, no indirect vs. direct evidence comparisons were made. Seven (pain intensity) and seven (depressive symptoms) pairwise comparisons were conducted. The corresponding network plots are depicted in Appendix 11. Appendix 12 displays sensitivity comparisons of all interventions and the usual care groups. With low certainty evidence, antidepressant therapy and pain self-management (pain and depressive symptoms), self-system therapy (pain and depressive symptoms), online- and mobile-based therapy (depressive symptoms), education (depressive symptoms) as well as acceptance and commitment therapy (depressive symptoms) were superior to usual care.

## Discussion

### Overall findings

Our systematic review with network meta-analysis provides first insights into the comparative effectiveness of various interventions with a substantial physical activity component for individuals with comorbid depressive symptoms and chronic LBP. We synthesized direct and indirect evidence from five RCTs including data from 834 participants and eight treatment categories, comparing a range of interventions targeting both physical and psychological outcomes. Unlike previous reviews, our study specifically required participants to have both clinically relevant depressive symptoms (meeting clinical cut-offs) and chronic LBP, which resulted in the inclusion of only five RCTs. For pain intensity and depressive symptoms, yoga with education demonstrated the most favourable and, for depressive symptoms, clinically relevant effects. These findings align with previous research highlighting the role of holistic interventions such as yoga, which integrates physical, cognitive, and emotional components, as an effective therapeutic approach for both LBP(^49^ for review) and depressive symptoms(^31^ for review). Of note, the certainty of evidence remains very low for the effects of yoga with education due to the small number of comparisons, underscoring the need for further robust, high-quality RCTs to confirm these findings.

### Depressive symptoms

Yoga combined with education led to a significant and clinically relevant reduction in depressive symptoms (mid-term) when compared to usual care, whereas other interventions – apart from the combination of antidepressant therapy and pain self-management – did not show clinically meaningful improvements over usual care conditions. Certainty of evidence also supports the clinically insignificant superiority of all other investigated interventions over usual care for the reduction of depressive symptoms. This finding should, however, be treated with caution due to the low small number of included studies and comparisons between different conditions.

The effectiveness of yoga was shown in recent reviews for depressive symptoms and LBP^31,49^ as separate conditions. The large effects shown in the present review (and thus higher effect size compared to recent meta-analyses^31^) on depressive symptoms may be explained by the, on the one hand, choice of control condition (i.e., pamphlet group) as the impact of exercise on depressive symptoms is known to vary depending on the comparator^14^. On the other hand, the population in the study investigating yoga differed from those in the other included studies. Notably, this was the only study where the mean levels of depressive symptoms and low back pain intensity were below the cutoff value defined in our sensitivity analyses.

The combination of antidepressant medication and pain self-management yielded moderate effects on depressive symptoms, aligning with findings from a recent network meta-analysis on medication^50^. The effects of self-management on depressive symptoms as a standalone intervention remain unclear, though first evidence from individuals with spinal cord injuries suggested potential benefits^51^. Prior research indicates that behavioural activation therapy approaches may not be more effective than control conditions^52^, aligning with our findings. While initial evidence (with low certainty) suggests that different forms of multimodal exercise therapy may improve depressive symptoms, further well-designed RCTs are needed to strengthen the current evidence.

### Pain, disability and quality of life

For pain reduction, yoga with education, antidepressant therapy with pain self-management as well as online- and mobile-based therapy demonstrated moderate but clinically non-relevant effects (mid-term). Cramer et al.^53^ reported in a meta-analysis moderate short- (SMD = -0.48) and smaller long-term effects (SMD = -0.33) of yoga interventions on pain intensity, further highlighting the potential of yoga for pain relief in patients. The low certainty evidence observed across all interventions highlights significant methodological limitations, such as a small sample size, heterogeneity, and reliance on indirect evidence for a majority of comparisons. The finding that antidepressant medication does not clinically improve pain compared to placebo aligns with systematic reviews on antidepressant use for LBP^54,55^. However, the type of medication may play a moderating role in pain outcomes^56^. Based on a small number of studies, tricyclic and tetracyclic antidepressants appear to produce moderate symptom reductions for patients with chronic LBP.

Previous studies have reported meaningful effects of motor control exercise interventions on disability^11^. The present review revealed that no intervention (except antidepressant therapy and pain self-management at long-term) reached clinically relevant effects on disability. With respect to quality of life, only two studies, i.e., online- and mobile-based therapy as well as antidepressants with pain self-management provided data for the long-term analyses. Both interventions showed small beneficial effects in favour of the interventional approaches. However, due to the small sample size, these findings should be interpreted with caution. Importantly, improving quality of life is often the primary goal for patients with LBP and depressive symptoms, extending beyond mere symptom reduction. Even small improvements may enhance social participation, emotional well-being, and work capacity. Notably, isolated studies have suggested rather moderate effects on quality of life, with SMDs of 0.39 for depression^57^ and a standardized mean effect size of 0.29–0.81 for LBP, depending on the specific dimension of quality of life assessed^58^. These discrepancies suggest that exercise may be less effective when LBP and depressive symptoms co-occur, that interventions may need to be better tailored, or that alternative exercise modalities, such as resistance training may have a greater impact on quality of life and should be further explored in future research. Given the societal impact of chronic pain and depressive symptoms, improving quality of life could also yield economic benefits by reducing disability and increasing work productivity^41^.

### Physiological and psychological mechanisms

Comorbid conditions, such as chronic pain and depressive symptoms, involve complex interactions between psychological and physiological mechanisms, which may reduce the effectiveness of single-target interventions. Pain and depression are closely linked and mutually reinforce each other^59^. The concept of allostasis has been suggested to explain that chronic stressors (e.g., persistent pain and depression) accumulate over time, increasing allostatic load and making individuals more susceptible to disease. To disrupt this cycle, comprehensive treatment approaches addressing all symptoms are essential^60^. Thus, for instance, antidepressant therapies may fail to address neuromuscular and inflammatory contributors to pain, while solely pain-focused treatments may lack the neurobiological impact necessary to alleviate depressive symptoms. Multimodal interventions, such as combined exercise and behavioural therapies, are crucial, as they may address both physical and psychological components. Notably, in multimodal programs, it is challenging to determine whether improvements in depressive symptoms result from the treatment itself or are merely a consequence of pain reduction, and vice versa^59^.

Beyond improving overall health^61,^exercise may exert disease specific benefits through neurobiological, anti-inflammatory, and neuromuscular processes. Exercise may enhance brain-derived neurotrophic factor (BDNF) and vascular endothelial growth factor (VEGF), both of which promote neurogenesis and neuroplasticity, crucial in the treatment of depressive symptoms^18^. Additionally, anti-inflammatory effects of exercise reduce systemic inflammation by downregulating pro-inflammatory cytokines such as IL-6 and TNF-α, which are linked to chronic pain and depression^18,62^. Furthermore, such interventions contribute to neuroendocrine changes, such as reduced cortisol response and improved sleep quality, which collectively enhance resilience to stress and mitigate chronic pain symptoms. Notably, relaxation and structured breathing exercises in yoga likely support autonomic balance, providing a pathway for both immediate and long-term relief from LBP and associated psychological distress^63^.

### Limitations and future studies

Several limitations of the included studies and of our review must be acknowledged. First, the certainty of evidence was predominantly rated as low or very low according to GRADE, indicating substantial uncertainty in the estimated effects and limiting confidence in the conclusions. The small number of included trials and participants constrains the generalizability of findings, and the inability to calculate heterogeneity and inconsistency metrics due to sample size limitations reduces the robustness of conclusions. These limitations are partly attributable to the strict inclusion criteria, which required participants to have both comorbid clinically relevant depressive symptoms and chronic LBP. Future reviews may consider broader inclusion criteria and include dedicated searches of grey literature databases to identify potentially unpublished studies and strengthen the evidence base. A key methodological issue is the lack of multiple direct comparisons of the interventions, which significantly weakens the reliability of indirect evidence and treatment rankings. Indeed, most comparisons were informed by single studies only, limiting the stability of effect estimates. Sparse network connectivity, evident in the limited number of direct comparisons (e.g., 32% for pain intensity and depressive symptoms), diminishes the strength of findings. Usual care conditions likely varied across studies, introducing additional heterogeneity that could not be formally quantified. Furthermore, including additional potential effect modifiers could have further supported the plausibility of the transitivity assumption.

Of note, we included studies where physical activity accounted for at least 33% of the intervention. Consequently, other components, such as behavioural or educational elements, might have contributed to the observed improvements in the depressive symptoms, pain, and disability^64,65^. It remains unclear whether a higher proportion or intensity of physical activity could have resulted in greater benefits. Therefore, conclusions should be interpreted as reflecting the effectiveness of interventions with a substantial physical activity component rather than exercise-only interventions. In one study^44^, the proportion of physical activity was estimated to meet the ≥ 33% criterion, as two of six modules focused on physical activity^48^. However, the actual exposure to physical activity may have varied between participants. Future studies should investigate the specific impact of exercise alone and in combination with other components to better understand its role in improving outcomes. Future research should prioritize well-designed interventions with appropriate long-term follow-ups, multicentre RCTs, and rigorous assessments of clinically relevant outcomes to build a more conclusive evidence base for the optimal management of chronic LBP and depressive symptoms. In general future trials are needed to develop interventions that address both depressive symptoms and LBP.

### Practical applications

While evidence and guidelines suggest to perform aerobic or strength training for depression^14,66^ and strength and motor control exercises for LBP^10,11^, a combined approach tailored to these comorbid conditions including educational parts may maximize therapeutic benefits^6,15,25,26^. Personalized approaches are crucial to meet the unique needs of patients with chronic LBP and depressive symptoms. Identifying whether pain or depressive symptoms are the primary “concern” could help prioritize interventions; for example, yoga may be particularly effective for both physical and psychological improvements, whereas alternative therapies may be preferable for depression-driven cases. Integrating psychological models, like the biopsychosocial approach^67,68^, ensures that biological, psychological, and social factors are considered. Tailored, widely accessible, scalable, and cost-effective interventions^64^, potentially combined with pharmacologicaloptimization where appropriate, may support patient-centred care for these complex comorbid conditions.

## Conclusion

This review highlights the potential of interventions incorporating a substantial physical activity component for managing comorbid LBP and depressive symptoms. Yoga in combination with education showed the most promise, particularly for depressive symptoms; however, these findings were derived from a single study and should therefore be interpreted with caution. The limited certainty of evidence and reliance on indirect comparisons emphasize the need for high-quality, well-designed studies. A personalized approach that prioritizes the dominant condition, i.e., pain or depressive symptoms, while integrating psychological and physical interventions may be crucial for optimizing outcomes and effectively addressing the multifaceted nature of these comorbid conditions.

## Data Availability

Data relevant to the study are included in the article or available in the supplementary files. All other relevant data can be found in a public repository: https://osf.io/bvx37.

## Declarations

### Study pre-registration statement

This systematic review with network meta-analysis was performed with an a priori defined protocol which is published in the PROSPERO database (ID: CRD42024523604).

### Equity, diversity, and inclusion statement

Secondary analyses; all relevant studies were included irrespective of the study population. Among the five authors, only one is female (not balanced). The distribution is balanced and consists of junior and senior researchers. All authors are from Germany.
